# Color Image Norms in Mandarin Chinese

**DOI:** 10.3389/fpsyg.2017.01880

**Published:** 2017-10-25

**Authors:** Dandan Zhou, Qi Chen

**Affiliations:** ^1^School of Psychology, South China Normal University, Guangzhou, China; ^2^Center for Studies of Psychological Application, South China Normal University, Guangzhou, China; ^3^Guangdong Key Laboratory of Mental Health and Cognitive Science, South China Normal University, Guangzhou, China

**Keywords:** Mandarin Chinese, color images, name agreement, familiarity, visual complexity, age of acquisition, viewpoint agreement

## Abstract

The present study comprises two parts, an object picture naming task and rating tasks, and reports naming latencies and norms for 435 color images in Mandarin Chinese. These norms include name agreement (%), *H*-value, concept agreement, familiarity, visual complexity, age of acquisition (AOA) based on adult ratings, object agreement, viewpoint agreement, word frequency, and word length. We examined correlations between the norms and explored the internal structure among these correlative variables by a factor analysis. Four factors were extracted, which accounted for 74.86% of the total variance. These data were analyzed to identify variables with significant contributions to naming latencies using multiple regression analysis, including norms of name agreement (%), familiarity, word frequency, concept agreement, AOA, and object agreement. These variables explained 54.70% of the total variance of naming latencies. This work presents a new set of photo stimuli and a large set of normalized variables. We expect that this study will provide useful materials for further researches.

## Introduction

Images of objects are essential materials in many fields of psychological research, such as visual perception, language, memory, and attention (Schiano and Watkins, 1981; Bonin et al., 2002; Goodale and Westwood, 2004; Filliter et al., 2005; Gomez and Shutter, 2008; Denkinger and Koutstaal, 2014). Studies of neural mechanisms and clinical research also use pictures to explore cognitive processes (Hirsh and Funnell, 1995; James et al., 2002; Hawco et al., 2013; van de Nieuwenhuijzen et al., 2013). However, some variables of these images are differ substantially, which can affect object recognition and naming. For instance, objects with high name agreement are named more quickly than low-name-agreement objects, because the lower the name agreement, the more names there are for one object (Vitkovitch and Tyrrell, 1995). A growing number of studies have investigated the contributions of the age of acquisition (AOA) of the object concept to word and picture identification (Juhasz, 2005). Furthermore, viewpoint agreement has been found to affect object identification and object recognition memory (Bülthoff and Newell, 2006; Gomez and Shutter, 2008). Larger deviations between the object viewpoint and the canonical viewpoint correspond to lower efficiency of object perception. In addition to name agreement, AOA, and viewpoint agreement, other variables that influence cognitive processes include visual complexity and concept familiarity. Researchers who wish to use these images in their studies should control for irrelevant variables of stimuli or systematically balance these variables across experimental conditions to facilitate an accurate and reasonable interpretation of the results. Therefore, to decrease the influence of image features and to explain experimental results appropriately, it is essential to normalize object images.

Previous research has primarily employed three types of standardized pictures: black-and-white line drawings, colorized versions of these line drawings, and color images (photographs). Black-and-white line drawings consist of hand drawings based on selected concepts. A pioneering normalization study of line drawings was conducted by Snodgrass and Vanderwart (1980). They presented a set of 260 black-and-white line drawings in English that were standardized on four key variables: name agreement, image agreement, familiarity, and visual complexity. Subsequently, several similar line-drawing normalization efforts have been pursued in different languages (Barry et al., 1997, for British; Bonin et al., 2003, for French; Cuetos et al., 1999, for Spanish; Liu et al., 2011, for Mandarin Chinese; Nishimoto et al., 2005, for Japanese; Pind et al., 2000, for Icelandic; Sirois et al., 2006, for Canadian French). These standardized sets of object images have been widely used in many research fields; they have even been used to compare the influence of different cultures on cognitive processes (Bates et al., 2003; Yoon et al., 2004; Łuniewska et al., 2016).

However, certain pictorial features, such as color or texture, are reduced in typical line drawings. Earlier studies have found that color is a useful cue for object processing (Price and Humphreys, 1989; Wurm et al., 1993; Tanaka and Presnell, 1999). Consistent with previous studies, Rossion and Pourtois (2004) found that adding color information could improve name agreement. Uttl et al. (2006) additionally found that objects with color were easier to identify than black-and-white photos. Therefore, to meet the need for color images in experiments, researchers added color and texture information to the original Snodgrass and Vanderwart (1980) pictures to produce norms for colorized versions of line drawings (Rossion and Pourtois, 2004). Subsequently, these colorized pictures were normalized by other researchers in different languages (Tsaparina et al., 2011; Bakhtiar et al., 2012; Bonin et al., 2013; Raman et al., 2014).

Although color and texture information make black-and-white line drawings much more similar to real-life objects, these pictures still lack ecological value. In particular, they cannot satisfy the requirements of experiments that aim to study real-life situations (Brodeur et al., 2010). For this reason, several investigators have considered collecting sets of color images (photos). In the early phase, researchers provided a small set of photographs of objects, which were downloaded from the web or other online sources (Viggiano et al., 2004; Adlington et al., 2009). In recent years, an increasing number of large sets of photo materials have been standardized in different languages (Brodeur et al., 2010, 2014; Moreno-Martínez and Montoro, 2012; Shao and Stiegert, 2016).

However, there are few normalized datasets of color images in Mandarin Chinese; most normalized images still consist of black-and white line drawings (Shu et al., 1989; Zhang and Yang, 2003; Liu et al., 2011). To our knowledge, only one study has reported normative data for colorized pictures in Chinese (Weekes et al., 2007). That study also suggested that color was an integral part of the object representation. However, the images used in that work were the colorized drawings of Snodgrass and Vanderwart (1980) produced by Rossion and Pourtois (2004), not real color images of objects. Thus, the problem of ecological validity may still exist.

Accordingly, the present work aimed to collect a large sample of color images of common objects and to normalize these images according to several important variables: name agreement, concept familiarity, visual complexity, AOA based on adult ratings, object agreement, and viewpoint agreement. These features were standardized due to their potential effects on cognitive processes.

## Materials and methods

### Materials

The procedure for obtaining the color images involved three main steps. First, we selected object concepts from the study of Liu et al. (2011). Several concepts appeared twice in their study. For instance, the English words “swan” and “goose” are both translated as “é” in Mandarin Chinese. In these cases, we distinguished the two concepts based on their English names (“swan” was “tiān é” and “goose” was “é” in Chinese pinyin). Through this process, we ultimately obtained 435 object concepts. Second, we collected color images of these concepts from different sources. Subsets of pictures were obtained from the original photographs' authors, including 62 from Brodeur et al. (2010) and 64 from Moreno-Martínez and Montoro (2012). The remainder was obtained via online sources. Most of the pictures were downloaded from websites such as http://image.baidu.com/ and http://cn.bing.com/images/. A small number were obtained from movie productions. All these images are intended solely for experimental purposes and noncommercial use. Third, we adjusted and edited the color images to ensure that they provided canonical views and maximal visual information. We then used Adobe Photoshop (Adobe Systems Inc., San Jose, US) to adjust the images, including applying shadows and filters as well as cropping around complex hair edges. Finally, the images were adjusted to approximately equal physical sizes and were positioned on a plain white background (400 × 300 pixels). All the color images are presented in the Supplementary Materials (File S1).

### Participants

A total of 191 Mandarin Chinese-speaking volunteers (105 females and 86 males; mean age = 20 years, ranging from 18 to 25 years) participated in this study. Different numbers of participants performed five different tasks. A group of 36 subjects (18 females) participated in the naming task; 39 (21 females) participated in estimating the age at which the concept of object was learned (AOA); 40 (23 females) participated in rating visual complexity; 38 (21 females) participated in rating concept familiarity; and 38 (22 females) participated in rating image agreement, including object agreement and viewpoint agreement. All participants were undergraduate or graduate students from universities in the Guangzhou area. They were all healthy, right-handed, and had normal or corrected-to-normal vision. After the experiment, the participants received modest monetary compensation for their participation.

This study was approved by the Human Research Ethics Committee for Non-Clinical Faculties, School of Psychology, South China Normal University. We obtained informed consent from all subjects before the experiments.

### Procedure

The experimental procedure comprised two parts: the color image-naming task and the rating tasks. Before each task, the instructions were explained in detail to the participants, who were encouraged to respond carefully. In each task, the participants first performed a short practice, followed by the experiment. All materials were randomly presented for every participant on microcomputers running E-Prime 2.0 (Psychology Software Tools).

#### Color image naming task

Participants were asked to name each image as briefly and accurately as possible by stating aloud the best and shortest name consisting of more than one Chinese character. The participants were asked to say “xiǎng bù qĭ” (“tip of the tongue,” TOT) if they were momentarily unable to remember the name, “bù zh$\bar{ı}$ dào” (“don't know name,” DKN) if they knew the object but not the name, and “bù rèn shi” (“don't know object,” DKO) if they had no idea what the object was (Brodeur et al., 2010). The responses were recorded on an audio recorder, and latencies were recorded using the PST-SRBOX (Psychology Software Tools, Inc., Serial Response Box). The procedure was similar to that of Moreno-Martínez and Montoro (2012). For each trial, a fixation cross (“+”) appeared in the center of the screen for 500 ms, followed by a blank interval of 500 ms. Then, the image was presented on the screen, where it remained for a maximum of 4,000 ms to ensure that the participant initiated a response before the image disappeared (Bates et al., 2003). The image disappeared as soon as a vocal response was made. If there was no response, the image disappeared automatically after 4,000 ms. The inter-trial interval was pseudo-randomized to a value between 1,000 and 2,000 ms. Every 50 trials, the participant was instructed to take a break; there were eight such short rest periods. The complete experimental session lasted approximately 45 min. All participants performed the naming task using the same computer in a quiet room.

#### Rating tasks

All rating tasks were performed using procedures described in previous studies (Liu et al., 2011; Moreno-Martínez and Montoro, 2012). For *age of acquisition* (AOA), participants were asked to decide the age at which the image concept was learned. *Visual complexity* was defined as “the amount of detail and intricacy of the color image”. The *familiarity* of image concepts was estimated by assessing “how usual or unusual exposure to this concept is in your life, including direct exposure (e.g., seeing a real object) and mediated exposure (e.g., seeing an object represented in a book)” (Adlington et al., 2009). Participants were told to rate the concept itself rather than the image. *Image agreement*, using the manipulation described by Brodeur et al. (2010), was divided into two variables: object agreement and viewpoint agreement. In this task, a single concept selected in this study was first presented. Participants had 5 s to imagine the object depicting this concept. Then, the corresponding color image appeared. For *object agreement*, the participants determined the extent to which the presented image corresponded to the mental image they had generated for the concept, not taking into account differences in orientation. For *viewpoint agreement*, participants were asked to judge the following: “How closely is the presented image positioned to what you imagined?” Half the subjects performed the object agreement task first and the viewpoint agreement task subsequently, and the task order was inverted for the other half. If participants could not generate a mental image or were not familiar with the concept, they could press zero.

All ratings, with the exception of AOA (7-point rating scale, 1 = 0–2 years, 2 = 3–4 years, 3 = 5–6 years, 4 = 7–8 years, 5 = 9–10 years, 6 = 11–12 years, and 7 = 13 years or older), were recorded on a 5-point rating scale that appeared on screen below the image. Participants were asked to press the corresponding number on the keyboard. Each rating task was divided into five blocks with a rest period between each block.

### Analyses

For the naming task, two recorders recorded naming responses separately. Failure to provide a verbal response, adjectival concepts, and indistinguishable responses were regarded as invalid naming responses. According to Bates et al. (2003), invalid response times included times for all invalid naming responses, coughs, hesitations, false starts, repetitions, prenominal verbalizations, or missing reaction times (RTs) (i.e., when a participant produced a name, but it failed to properly trigger the voice key). One participant was excluded because he did not name the images as briefly and accurately as possible. Four other participants were excluded from further analyses due to their high rates of invalid responses (above 15%).

*Name agreement* was measured in two ways: the information statistic *H* and the percentage of participants giving the most common and correct name (dominant name). When computing *H*-values, TOTs, DKNs, DKOs, and invalid naming responses were eliminated, but these trials were included when computing percentage agreement scores. The index *H-*value is considered more informative than name agreement (Moreno-Martínez and Montoro, 2012). *H* was calculated for each picture using the following formula (Snodgrass and Vanderwart, 1980):

$$\begin{array}{l} H=\overset{k}{\underset{i=1}{\sum }}P_{i}log_{2}\left(1/P_{i}\right) \end{array}$$

where *k* is the number of names given for a color image, and *p*_*i*_ is the proportion of subjects providing each unique name. *H* = 0 represents perfect agreement among participants (i.e., only one name), and the *H*-value increases as agreement decreases.

According to the dominant name, *naming latency* (RT) was calculated for each picture. To eliminate the influence of outliers, scores falling beyond 2.5 SD of the grand mean RT were removed (2.88%). Additionally, we selected *word frequency* from the SUBTLEX-CH (Cai and Brysbaert, 2010) in log-transformed form. Ten dominant names were eliminated because their word frequencies were unavailable in this corpus.

## Results

Table 1 provides norms for the 435 color images on each variable. For the naming task, we included the naming latency as the dependent variable, word frequency and the two measures of name agreement: *H*-value and percentage name agreement. In addition, concept agreement was calculated according to the proportion of participants who provided the correct concept, the word length of the dominant name was calculated in numbers of characters, and the target name agreement was calculated based on the concepts from Liu et al. (2011). The results of each rating task are listed below. Skewness values for the features are provided to show their distribution. All the standardized variables for each item are provided in the Supplementary Materials (Data Sheet 1).

**Table 1:** Descriptive statistics for the 435 color images.

**Variable**	**Code**	**Mean**	**SD**	**Min**.	**Max**.	**Skewness**
Naming latency	RT	1039	179	665	1660	0.53
Name agreement (%)	NA%	0.68	0.25	0.03	1.00	−0.64
*H*-value	*H*	1.05	0.77	0.00	3.18	0.51
Concept agreement	CA	0.85	0.19	0.03	1.00	−1.71
Word length	W_len	1.99	0.56	1.00	4.00	0.08
Word frequency	W_fre	2.40	0.75	0.00	4.47	−0.02
Age of acquisition	AOA	3.76	0.83	2.13	6.13	0.28
Familiarity	Fam	3.86	0.59	2.00	4.82	−0.49
Object agreement	OA	3.67	0.53	1.68	4.68	−0.59
Viewpoint agreement	VA	3.46	0.59	2.03	4.71	−0.02
Visual complex	VC	2.99	0.87	1.05	4.70	−0.08
Tip of tongue (%)	TOT	0.02	0.05	0.00	0.39	2.91
Don't know name (%)	DKN	0.02	0.05	0.00	0.26	2.52
Don't know object (%)	DKO	0.02	0.06	0.00	0.61	4.93

*Naming latency was measured in milliseconds. Word length is given in number of characters. Word frequency is expressed in log-transformed values obtained from the SUBTLEX-CH database (Cai and Brysbaert, 2010)*.

As in previous normalization studies, after computing descriptive statistics, we performed a correlation analysis to examine how the norms were related to each other. Correlations between the norms are presented in Table 2. The data showed that all variables except visual complexity were related to naming latencies. Visual complexity was significantly related to only familiarity (*r* = −0.348, *p* < 0.01) and word frequency (*r* = 0.146, *p* < 0.01). The two measures of name agreement were highly correlated (*r* = −0.912, *p* < 0.01), and the two measures of image agreement were significantly correlated (*r* = 0.306, *p* < 0.01).

**Table 2:** Matrix of pairwise correlations between all variables.

**Variable**	**RT**	**NA%**	***H***	**CA**	**W_len**	**W_fre**	**AOA**	**Fam**	**OA**	**VA**
NA%	−0.614^**^									
*H*	0.519^**^	−0.912^**^								
CA	−0.621^**^	0.740^**^	−0.582^**^							
W_len	0.171^**^	−0.083	0.053	−0.054						
W_fre	−420^**^	0.339^**^	−0.244^**^	0.322^**^	−0.443^**^					
AOA	0.504^**^	−0.410^**^	0.325^**^	−0.469^**^	0.278^**^	−0.406^**^				
Fam	−0.517^**^	0.365^**^	−0.288^**^	0.529^**^	0.054	0.245^**^	−0.482^**^			
OA	−0.260^**^	0.293^**^	0.236^**^	0.304^*^	0.103^*^	−0.113^*^	0.023	0.261^**^		
VA	−0.132^**^	0.112^*^	−0.072	0.147^**^	0.064	0.094	−0.038	0.206^**^	0.306^**^	
VC	0.019	0.092	−0.077	0.026	−0.025	0.146^**^	0.044	−0.348^**^	−0.040	−0.037

* p < 0.05;

** *p < 0.01 (two-tailed). Variable abbreviations are the same as in Table 1*.

The correlation analysis indicated that most variables had highly significant correlations with each other (see Table 2). We then performed factor analysis to explore the internal structure of ten correlated variables; the results are shown in Table 3. The KMO was.661, and Bartlett's test of sphericity produced a result of χ^2^(45) = 1967.131, *p* < 0.001, indicating that the factor analysis were effective for the correlated variables. Four factors were extracted from the ten variables. The first factor, *name agreement*, comprises the percentage name agreement, *H-*value, and concept agreement. Prior studies have found that the effect of AOA may occur at the lemma level (Belke et al., 2005). In the present study, we found that AOA, word frequency, and word length loaded highly on the second factor, *lexicon*. Familiarity and visual complexity loaded highly on only the third factor, *difficulty of object processing*, since visual complexity probably reflects image recognition (Weekes et al., 2007), and several studies have shown that familiarity correlates significantly with visual complexity (Shu et al., 1989; Zhang and Yang, 2003; Weekes et al., 2007; Liu et al., 2011). The last factor, *image agreement*, consists of object agreement and viewpoint agreement (Brodeur et al., 2010). Altogether, these components accounted for 74.86% of the total variance.

**Table 3:** Rotated loading of ten variables on four factors.

**Variable**	**Rotated factor loading**			
	**Factor 1**	**Factor 2**	**Factor 3**	**Factor 4**
NA%	0.950	−0.107	−0.008	0.103
H	−0.905	0.018	−0.069	−0.035
CA	0.805	−0.168	−0.215	0.168
AOA	−0.439	0.542	0.394	0.094
W_fre	0.263	−0.811	0.041	0.055
W_len	0.080	0.794	−0.050	0.070
Fam	0.414	−0.148	−0.728	0.229
VC	0.153	−0.137	0.845	0.013
OA	0.307	0.273	−0.070	0.668
VA	−0.023	−0.116	−0.046	0.889
Eigenvalue	3.462	1.664	1.318	1.042
% Variance	29.289	17.493	14.621	13.452

*Variable abbreviations are the same as in Table 1*.

For the 10 important variables, we first used simultaneous multiple regression analysis to identify variables with significant effects on naming latency. However, we found that the percentage name agreement showed a multi-collinearity problem (variance inflation factor, VIF, was greater than 10; tolerance value was 0.094). When the largest VIF (the reverse of tolerance) is in excess of 10, there may be multi-collinearity in the regression model (O'Brien, 2007). We then used stepwise multiple regression analysis to identify the predicting effect of variables on naming latency; the results are shown in Table 4. The VIF and tolerance values suggested that multi-collinearity had little effect on the regression model. The regression analysis yielded an adjusted *R*^2^ = 0.547, *F*_(6, 417)_ = 86.290, and *p* < 0.001. Name agreement (%), familiarity, word frequency, concept agreement, AOA and object agreement showed significant contributions to naming latencies. No other variables were significant.

**Table 4:** Stepwise multiple regression analysis on naming latency.

**Variable**	**Beta**	***t-*value**	**Tolerance**	**VIF**
NA%	−0.236	−4.670^***^	0.417	2.396
Familiarity	−0.215	−5.178^***^	0.619	1.615
W_fre	−0.195	−5.244^***^	0.771	1.297
CA	−0.162	−2.995^**^	0.367	2.772
AOA	0.160	3.844^***^	0.620	1.613
OA	−0.122	−3.318^**^	0.791	1.265
H	0.070	0.805	0.140	7.144
W_len	0.045	1.192	0.734	1.362
VA	0.013	0.385	0.878	1.139
VC	0.000	−0.008	0.777	1.287

** p < 0.01;

*** *p < 0.001. VIF, variance inflation factor. Variable abbreviations are the same as in Table 1*.

On the basis of the naming latencies, pictures were divided into five difficulty levels with 87 items in each level. The mean value of all the variables in each level is shown in Table 5. In addition to visual complexity, most variables showed clear increasing or decreasing trends accompanying increases in naming latencies. These measures will provide substantial additional information for future studies of cognitive processes.

**Table 5:** Mean values of all variables by level.

**Level**	**RT**	**NA%**	***H***	**CA**	**Fam**	**VC**	**AOA**	**OA**	**VA**	**W_len**	**W_fre**
1	815	0.86	0.55	0.87	4.25	2.95	3.22	3.88	3.55	1.87	2.75
2	930	0.78	0.80	0.87	4.10	2.94	3.43	3.69	3.59	1.89	2.60
3	1014	0.72	1.00	0.85	3.84	2.98	3.72	3.65	3.39	1.98	2.57
4	1124	0.63	1.25	0.82	3.73	3.10	4.04	3.74	3.39	2.09	2.18
5	1316	0.43	1.64	0.83	3.37	2.97	4.39	3.40	3.38	2.14	1.89

*On the basis of the naming latencies, pictures were divided into five difficulty levels with 87 items in each level. Variable abbreviations are the same as in Table 1*.

## Discussion

This study is the first to provide norms for a large set of color images in Mandarin Chinese, and it standardized several important variables: name agreement (*H*-value and the percentage of dominant name), concept agreement, word frequency, word length, AOA, familiarity, image agreement (object agreement and viewpoint agreement), and visual complexity. We examined correlations between the norms and explored the internal structure among these correlative variables by a factor analysis. Four factors were extracted and accounted for a large portion of the total variance (74.86%). Furthermore, we used multiple regression analysis to identify variables with significant contributions to naming latencies, including norms of name agreement, familiarity, word frequency, concept agreement, AOA, and object agreement. These variables explained 54.70% of the total variance of naming latencies.

Our main purpose was to establish Mandarin Chinese norms for photo stimuli. Since most of the operations in the current study were conducted with reference to previous studies, we compared our norms with these studies to establish the validity for the current study. First, we compared the results with other Chinese studies (Zhang and Yang, 2003; Weekes et al., 2007; Liu et al., 2011). A comparison of the traditional line drawings (Liu et al., 2011) and the color images used in the present study (Table 6) revealed very small differences for all the common variables. Except for RT, percentage name agreement and concept agreement (*p* > 0.05), statistically significant differences were observed on other variables (*p* < 0.001). However, it is important to test the reliability of the differences across different studies, we conducted a correlation analysis on values of the overlapping variables to test whether the items elicits similar responses. We found that all these variables were significantly related to each other; the Pearson's correlations are presented in Table 7. The absolute values of the correlation coefficients were all high, suggesting that the items of the current study elicited similar response patterns to that of line drawings (Liu et al., 2011). The *H*-values in the current study were lower than those reported by Liu et al. (2011), which may suggest that color images have a more concentrated distribution of names.

**Table 6:** Comparison between norms from the present study and norms for line drawings (Liu et al., 2011).

		**RT**	***H***	**NA%**	**CA**	**Fam**	**VC**	**AOA**
Present study	Mean	1039	1.05	0.68	0.85	3.86	2.99	3.76
	SD	179	0.77	0.25	0.19	0.59	0.87	0.83
Liu et al. (2011)	Mean	1044	1.32	0.66	0.86	4.36	2.81	3.44
	SD	210	0.84	0.23	0.16	0.47	0.84	1.15

Variable abbreviations are the same as in Table 1

**Table 7:** Correlations between present study and studies of Liu et al. (2011), Brodeur et al. (2010), Moreno-Martínez and Montoro (2012), for the overlapping norms.

	**Liu et al., 2011; 435 items**	**Brodeur et al., 2010; 62 items**	**Moreno-Martínez and Montoro, 2012; 64 items**
RT	0.579^**^	–	–
*H*	0.668^**^	0.358^**^	0.370^**^
NA%	0.660^**^	–	–
CA	0.509^**^	–	–
Fam	0.585^**^	0.429^**^	0.811^**^
VC	0.641^**^	0.858^**^	0.876^**^
AOA	0.858^**^	–	0.585^**^
OA	–	0.594^**^	–
VA	–	0.547^**^	–

** *p < 0.01. –, not computed. Variable abbreviations are the same as in Table 1*.

Moreover, consistent with the results of Liu et al. (2011), most variables in the present study had significant correlations. We conducted factor analysis to provide more information about the internal structure of the correlated variables. Four factors were extracted from the ten variables: name agreement, lexical attributes of associated words, difficulty of image processing, and image agreement. The regression analysis further showed that familiarity, AOA, concept agreement, name agreement (%) and image agreement had a significant effect on naming latencies, which were strikingly similar to those reported by Liu et al. (2011). Weekes et al. (2007) also reported that name agreement (%), familiarity, AOA made independent contributions to naming RTs. The two most reliable predictors were name agreement and rated AOA, which were well documented for line drawings in different languages (Barry et al., 1997; Cuetos et al., 1999; Bonin et al., 2003; Nishimoto et al., 2005). In addition, we found that word frequency (log-transformed form, obtained from the SUBTLEX-CH dataset) had a significant impact on reaction time, which were in line with Zhang and Yang's study (2003) and other language studies (Barry et al., 1997; Cuetos et al., 1999). However, in Liu et al.'s study (Liu et al., 2011), the subjective word frequency had no significant effect on naming latencies. Instead of the subjective word frequency, we used the database of SUBTLEX-CH (Cai and Brysbaert, 2010) for word frequency (in log-transformed form), together with other variables in Liu et al.' study (Liu et al., 2011), to perform the simultaneous multiple regression analysis on naming RTs again. The total adjusted *R*^2^ = 0.671, *F*_(10, 423)_ = 87.425, and *p* < 0.001. The results were consistent with the findings of Liu et al. (2011). Concept familiarity, objective AOA, rated AOA, concept agreement, and image agreement showed significant contributions to naming RTs, which were in the decreasing order of standardized beta coefficients. For the percentage of name agreement, there was a marginal significant (*t* = −1.867, *p* = 0.063). However, we found that word frequency had a significant contribution to naming latencies (*t* = −2.558, *p* < 0.05).

Previous studies have suggested that concept familiarity is equivalent to word frequency and that higher frequency corresponds to greater familiarity (Ellis and Morrison, 1998; Cuetos et al., 1999). These two variables may have the same effect on picture naming (Johnson et al., 1996; Liu et al., 2011). However, contrary to this view, some studies have suggested that they are very different. Almeida et al. (2007) found that familiarity affected object identification, whereas Jescheniak and Levelt (1994) found that word frequency affected phonological lexicon access. Moreover, Graves et al. (2007) revealed that activity in the left posterior superior temporal gyrus modulated by word frequency but not concept familiarity. Bates et al. (2003) investigated the object-naming norms for line drawings in seven languages and found that word frequency had large effects on naming latencies for all the languages. Moreover, in our study, factor analysis suggested that word frequency and familiarity were explained by different dimensions of the stimuli's characteristics. Therefore, as with other varibles (Name agreement, familiarity, concept agreement, AOA and object agreement), word frequency influenced the speed of picture naming.

For the colorized versions of line drawings, Bakhtiar et al. (2012) and Bonin et al. (2013) reported that name agreement, image agreement, and AOA norms made significant independent contributions to naming latencies. Furthermore, they found that objective word frequency was reliable in object-naming latencies. However, Bonin et al. (2013) found that subjective word frequency was no significant in object naming. For normalized photos of objects, Shao and Stiegert (2016) found that naming latencies were significantly predicted by word frequency, which obtained from the SUBTLEX-NL database in log-transformed form (Keuleers et al., 2010). Therefore, we suggest that the different operations of word frequency might affect its independent effects on picture naming. Objective word frequency might be more effective than subjective frequency.

Lastly, we compared the norms of the present study with other normative studies for color images, especially for those of Brodeur et al. (2010) and Moreno-Martínez and Montoro (2012). We compared the norms of the current study with those of Brodeur et al. (2010) for overlapping items (62 color images), which are shown in Table 8. Although common variables were normalized in different languages (Mandarin Chinese and English), only small differences between them were observed. However, a comparison of our data and those of Moreno-Martínez and Montoro (2012) for the 64 color images in Spanish (Table 9) reveals large differences in *H*-values and AOA. We conducted correlation analysis to explore the different values of variables across these studies and found that all these variables were significantly related to each other (see Table 7). Although the correlation coefficients for the *H*-value were both relatively low (*r* = 0.358, *p* < 0.01; *r* = 0.370, *p* < 0.01), the significant relations also suggest that the items of the current study elicited similar response patterns to those of Brodeur et al. (2010) and Moreno-Martínez and Montoro (2012). The higher *H*-value in the present study suggests that the given object has more possible names in Mandarin Chinese than in English and Spanish (Liu et al., 2011).

**Table 8:** Comparison between the present study and Brodeur et al. (2010) for the 62 overlapping items.

		***H***	**Fam**	**VC**	**OA**	**VA**
Present study	Mean	1.09	3.92	2.78	3.62	3.49
	SD	0.83	0.66	0.83	0.55	0.58
Brodeur et al. (2010)	Mean	0.92	4.22	2.5	4.12	3.72
	SD	0.77	0.36	0.48	0.47	0.42

*Variable abbreviations are the same as in Table 1*.

**Table 9:** Comparison between the present study and Moreno-Martínez and Montoro (2012) for the 64 overlapping items.

		***H***	**Fam**	**VC**	**AOA**
Present study	Mean	1.02	3.94	3.03	3.44
	SD	0.79	0.59	0.80	0.83
Moreno-Martínez and Montoro (2012)	Mean	0.41	4.02	2.42	2.75
	SD	0.78	0.78	0.72	1.03

*Variable abbreviations are the same as in Table 1*.

In summary, this is the first study to report norms for 435 color images in Mandarin Chinese and to normalize these images according to the following important variables: name agreement (*H*-value and percentage name agreement), concept agreement, word frequency, word length, AOA, familiarity, image agreement (object agreement and viewpoint agreement), and visual complexity. Sets of line drawings and sets of our color images could become complementary tools for experimental research. We expect that this work will enhance the usefulness of color images as materials for cognitive and psycholinguistic research. In combination with other existing sets of photos, we hope that this study will provide useful materials for further research across cultures.

## Author contributions

QC conceived and supervised the experiments; QC and DZ designed the experiments; DZ implemented the experiments and collected data; DZ analyzed the results; and all the authors wrote and revised the manuscript.

### Conflict of interest statement

The authors declare that the research was conducted in the absence of any commercial or financial relationships that could be construed as a potential conflict of interest.

## Supplementary material

Data Sheet 1 Norming data for 435 color images (Excel).

File S1 Color images of the 435 items (RAR), including subsets of 62 pictures (in File 1) and 63 pictures (File 2). All the color images can be downloaded from the website http://pan.baidu.com/s/1c1QcDRa.
